# Concurrent uveitis in monozygotic twins in the context of systemic sarcoidosis

**DOI:** 10.1186/s12348-025-00504-7

**Published:** 2025-05-30

**Authors:** Doaa Maamoun Ashour, Reem Mohsen, Rahma A. Elziaty, Omnia Bahaa Attia, Haytham Samy Diab, Caroline Atef Tawfik

**Affiliations:** 1 Department of Ophthalmology, Faculty of Medicine, Ain Shams University, Cairo, Egypt; 2 Rheumatology Division, Department of Internal Medicine, Faculty of Medicine, Ain Shams University, Cairo, Egypt; 3 Pulmonary Medicine and Interventional Pulmonology, Faculty of Medicine, Ain Shams University, Cairo, Egypt

**Keywords:** Uveitis in twins, Ocular sarcoidosis, Familial uveitis, Concurrent uveitis

## Abstract

**Purpose:**

To report a case of two monozygotic twins presenting with simultaneous onset of bilateral uveitis of variable phenotypic presentations, one of whom was pathologically confirmed to have sarcoidosis.

**Observation:**

Two 21-year-old monozygotic male twins (Twin A and Twin B) presented with bilateral red eyes and photophobia of six weeks’ duration. Their past medical history included learning difficulties and low IQ since early childhood. Twin A was operated for Celiac Artery Compression Syndrome. Examination and work-up revealed that Twin A had bilateral anterior granulomatous uveitis, and retinal phlebitis in addition to hilar and mediastinal lymphadenopathy. A biopsy was taken, and histopathological examination showed non-caseating granuloma. Twin B had bilateral non-granulomatous anterior uveitis, chorioretinal lesions, and peripheral retinal vasculitis. Genetic testing in the form of a Whole Exome Sequencing was done, and no causal variant was detected for uveitis or sarcoidosis, however, a homozygous likely pathogenic duplication in *SYNGAP1* was detected. This mutation is associated with autosomal dominant intellectual developmental disorder.

**Conclusion:**

This is the first-reported case of concurrent bilateral uveitis in monozygotic twins, with confirmed sarcoidosis in one. This presentation highlights the role of genetic predisposition and shared environmental factors in disease onset and clinical manifestations. Further research into the genetic-environmental interplay is needed to elucidate the mechanisms underlying simultaneous disease onset and guide personalized monitoring strategies for at-risk families.

## Introduction

Uveitis is defined as intraocular inflammation with a wide range of etiologies, broadly classified into infectious and non-infectious categories. Although the precise pathogenesis of non-infectious uveitis remains incompletely understood, it is generally attributed to an interplay between specific genetic predispositions and dysregulation of immune system mechanisms [1, 2].

Familial occurrences of uveitis have been infrequently documented, with reports indicating diverse etiologies and varying anatomical presentations. These include associations with juvenile idiopathic arthritis, tubulointerstitial nephritis, sarcoidosis, Vogt-Koyanagi-Harada syndrome, and idiopathic uveitis [3–6]. Notably, idiopathic uveitis has also been reported in monozygotic twins, suggesting potential genetic or shared environmental factors [7]. 

Sarcoidosis is a multisystem inflammatory disease of unknown etiology, characterized by the presence of non-caseating granulomas. The incidence of sarcoidosis differs significantly across the world, likely due to variations in environmental exposures, case-surveillance practices, predisposing HLA alleles, and other genetic factors [8]. Reports of familial clustering and disease occurrence in twins suggest a genetic predisposition [9]. Ocular involvement, which can affect any part of the eye and its adnexal tissues, occurs in 25–70% of sarcoidosis patients and may be the initial manifestation of the disease in 20–40% of cases [10, 11]. Yang et al. reported cases of familial sarcoidosis with uveitis in two sisters in Taiwan [6]. Here, we present two monozygotic twins with uveitis, one of whom was pathologically confirmed to have sarcoidosis. To the best of our knowledge, this is the first reported case of simultaneous onset of uveitis associated with sarcoidosis in monozygotic twins.

## Case report

Two 21-year-old monozygotic male twins were referred to the uveitis clinic at Ain Shams University Hospitals, both presenting with bilateral red eyes and photophobia of six weeks’ duration. Both were diagnosed with learning difficulties and low IQ since early childhood. They work at a garment factory.

### Twin A

**Fig. 1:**
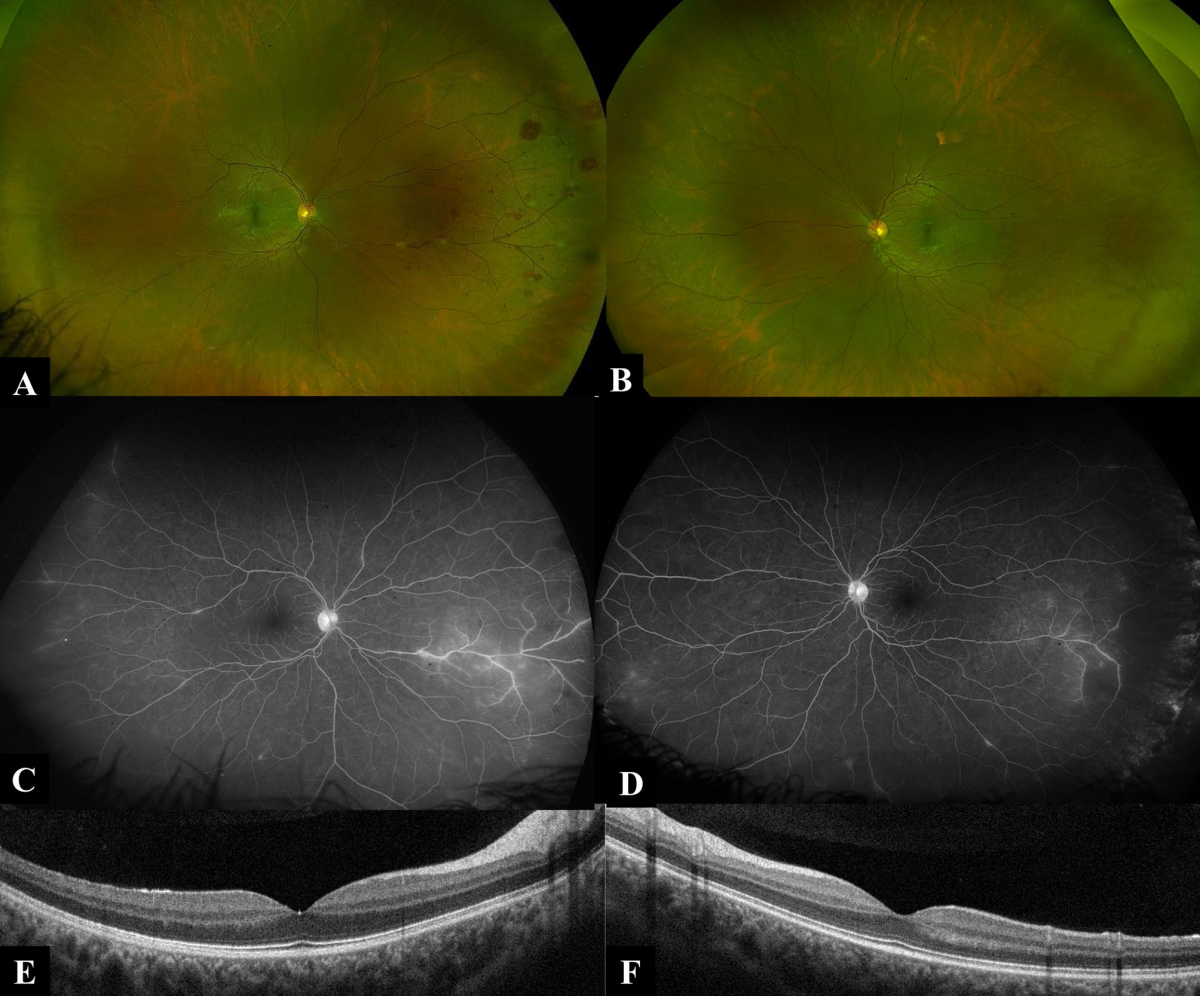
Ultra-wide fundus imaging and OCT of Twin A: **A** Ultra-wide field pseudocolor fundus image (Optos) of twin A right eye demonstrating nasally distributed intraretinal hemorrhages and perivascular inflammatory exudates (skip lesions). **B** Ultra-wide field pseudocolor fundus image (Optos) of twin A left eye demonstrating no evident choroidal, retinal or vascular lesions. **C** and **D** Ultra-wide field fundus fluorescein angiography late frames (Optos) of twin A right and left eye respectively demonstrating nasal and temporal mid-peripheral occlusive retinal periphlebitis associated with hot optic discs. **E** and **F** OCT of the macula of twin A right and left eye respectively demonstrating dry inner retinal layers with intact outer retinal layers, normal choroidal architecture and preserved foveal contours

Twin A presented with bilateral red eye and photophobia. Details of ocular examination are shown in Table 1. His past medical history included long history of gastrointestinal manifestations including discomfort, pain and vomiting related to meals with a recent surgery for Celiac Artery Compression Syndrome (also known as median arcuate ligament syndrome). During the general examination and system review, he was found to have back pain and multiple skin lesions on his back. Topical steroids and cycloplegics were prescribed and work-up was ordered. Laboratory work up showed negative Quantiferone TB Gold test, Syphilis serology, Toxoplasma serology, viral markers, and calprotectin in stool. Full blood count showed absolute lymphopenia (0.8* 10^3^, normal 1.5-4* 10^3^). ESR and CRP were mildly elevated. Chest X-ray was normal. Two weeks later, anterior segment inflammation showed insignificant improvement, thus systemic steroids were started. Later, fundus examination of the left eye revealed new onset retinal vasculitis with segmental appearance. Fundus Fluorescein Angiography (FFA) and Optical coherence Tomography (OCT) were ordered, results are shown in Fig. 1. Further work up was requested and revealed mildly elevated serum ACE (65.4, normal 13–63), negative ANA, ANCA-c, ANCA-p. Punch biopsy was taken from the skin lesions in his back and revealed mildly thickened collagen fibers without significant pathological changes. High resolution CT chest was ordered and revealed bilateral upper lobar tiny nodular infiltration with perilymphatic distribution associated with hilar and mediastinal lymphadenopathies. The patient was referred for further assessment by an expert pulmonologist, who arranged for bronchoscope and lymph node biopsy. Histopathological examination of the lymph node biopsy showed non caseating granulomatous inflammation suggestive of sarcoidosis.

**Table 1:** Findings of ophthalmological examination at the initial presentation

	Twin A		Twin B	
	OD	OS	OD	OS
CDVA	0.7	0.7	1.00	0.7
Refraction	-2/-0.5* 155	-2.25	-2.25/-0.75*160	-1/-1*20
KPs	Medium sized granulomatous white KPs		Fine white KPs	
Cells	+ 2	+ 2	+ 3	+ 2
Flare	+ 2	+ 2	+ 2	+ 2
IOP	16	18	12	14
Fundus	Unremarkable		Punched out well circumscribed choroidal lesion	

### Twin B

**Fig. 2:**
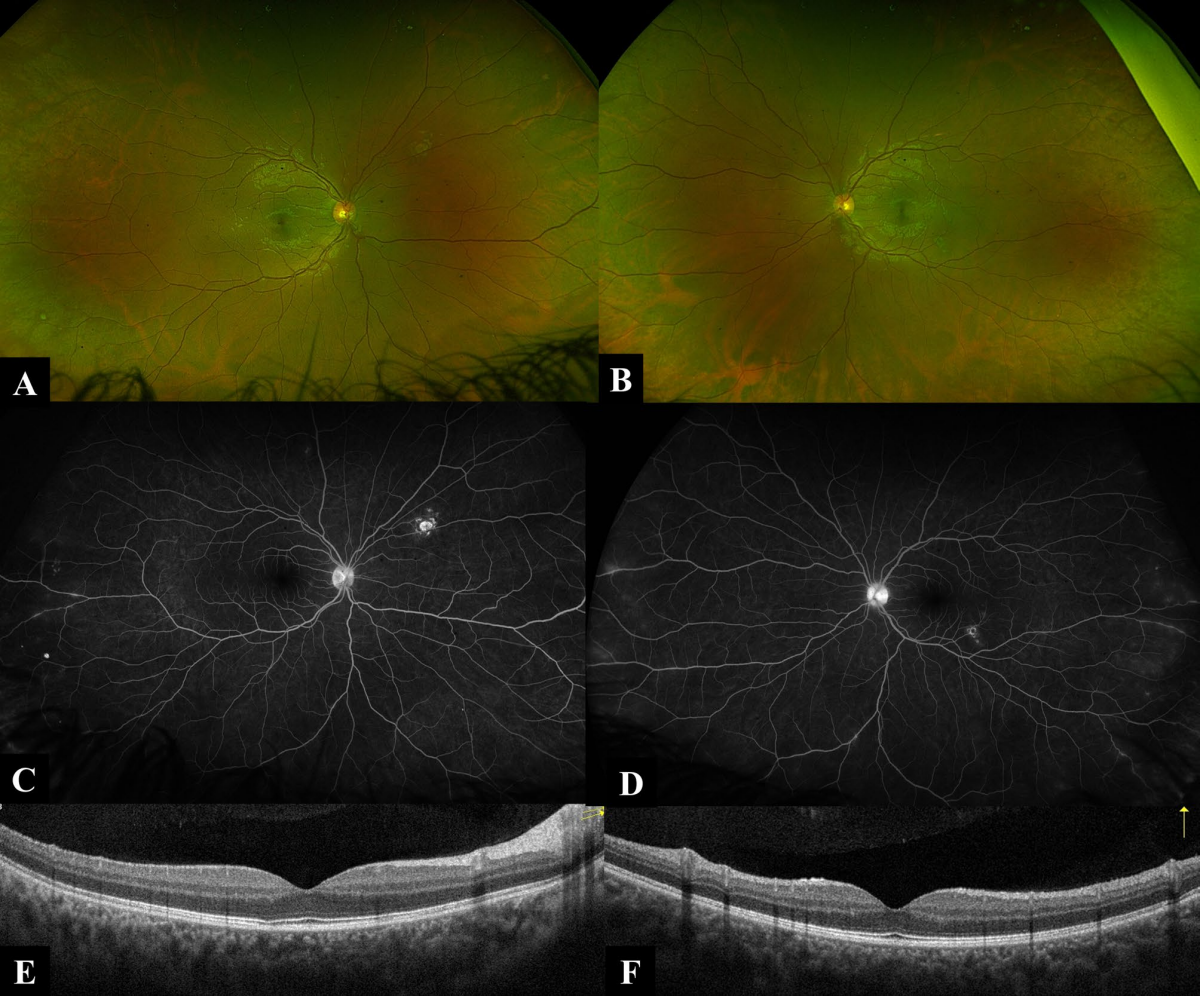
Ultra-wide fundus imaging and OCT of Twin B: **A** Ultra-wide field pseudocolor fundus image (Optos) of twin B right eye demonstrating superior nasal mid-peripheral punched out choroidal lesion. **B** Ultra-wide field pseudocolor fundus image (Optos) of twin B left eye demonstrating inferior temporal posterior pole punched out choroidal lesion. **C** and **D** Ultra-wide field fundus fluorescein angiography late frames (Optos) of twin B right and left eye respectively demonstrating nasal and temporal mid-peripheral retinal periphlebitis associated with hot optic discs and minimal leakage of the choroidal lesions. **E** and **F** OCT of the macula of twin A right and left eye respectively demonstrating dry inner retinal layers with intact outer retinal layers, normal choroidal architecture and preserved foveal contours

Similar to his twin brother, he presented with bilateral red eye, blurring, and photophobia. Ophthalmological examination results are shown in Table 1. His past medical history was unremarkable apart from the learning difficulties and low IQ. Same work-up was done as his Twin, however, all results turned out to be negative. He complained of back pain, MRI showed mild sacroiliitis. OCT, colored fundus photo, FAF and FFA were done (Fig. 2). Multimodal imaging delineated old choroidal lesions and revealed peripheral vascular leakage.

### Treatment received

Topical steroids and cycloplegics were started first. Mild improvement was observed in the anterior segment reaction; thus, systemic steroids (prednisolone 40 mg initially) were added after the initial laboratory work up.

During the diagnostic process, we initiated conventional immunosuppressive therapy (azathioprine) in addition to the previously prescribed systemic steroids and topical treatment.

For Twin A, anterior chamber inflammation fluctuated between + 1 and + 2 cells after three months of conventional therapy, and retinal vasculitis had progressed to the lower macular area, manifesting as perivascular exudates and hemorrhage. For Twin B, while initial control of anterior segment inflammation was achieved, a flare-up occurred when the oral steroid dose was reduced to 25 mg/day. Due to inadequate response to conventional therapy in both cases, treatment escalation was deemed necessary.

After counseling and discussing available options -either adding or shifting to another conventional immunosuppressant or transitioning to biologic therapy-adalimumab biosimilar (40 mg subcutaneous injection every two weeks) was initiated. Both twins showed gradual improvement, allowing for stepwise tapering of topical and systemic steroids.

**Fig. 3:**
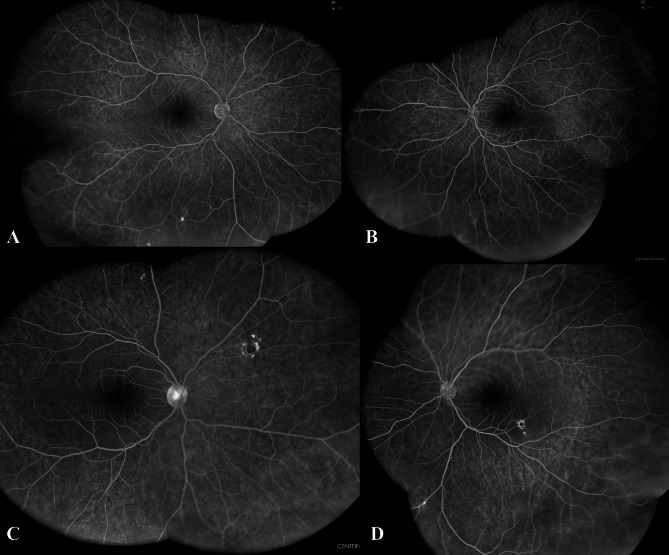
Ultra-wide field fundus imaging of Twin A and Twin B after 18 months of treatment: **A** and **B** Ultra-wide field fundus fluorescein angiography late frames (EIDON) of twin A right and left eye respectively demonstrating regressed retinal vasculitis with re-perfusion of the peripheral retina. **C** and **D** Ultra-wide field fundus fluorescein angiography late frames (EIDON) of twin B right and left eye respectively demonstrating regressed retinal vasculitis and late edge staining of the punched-out choroidal lesions

Follow-up fundus fluorescein angiography was done at 18 months post-treatment (Fig. 3) demonstrated near-complete resolution of the retinal vasculitis. Over a two-year follow-up period, both patients have maintained stable disease without recurrence.

### Genetic testing

Based on suspicions that these twins may be suffering from late-onset Blau syndrome due to the aggregation of cutaneous manifestations, arthritis and uveitis, we performed whole-exome sequencing (WES) on an Illumina platform by using genomic DNA extracted from a peripheral blood sample. WES did not reveal a clear causal variant despite focusing on diseases of autoimmunity, immune dysregulation as well as diseases that cause uveitis. Specifically, no variants in *NOD2* gene associated with Blau syndrome were identified. On another note, WES did reveal a novel, homozygous likely pathogenic duplication in *SYNGAP1*: c.14_16 which is associated with autosomal dominant intellectual developmental disorder-5 (OMIM # 612621).

## Discussion

Uveitis is a multifactorial condition with variable etiologies, including infections, immune-mediated diseases, and masquerade syndromes secondary to malignancies or other non-neoplastic disorders [12, 13]. 

Based on the phenotypic presentation of uveitis in the twins, we needed first to establish a differential diagnosis and to initiate systematic investigations guided by their clinical features. As per their ocular condition, Twin A had bilateral anterior granulomatous uveitis, and retinal vasculitis -mainly phlebitis with segmental appearance-, while Twin B presented with bilateral non granulomatous anterior uveitis, few chorioretinal lesions, and peripheral retinal vasculitis. Despite discernible phenotypic divergence in the twins’ clinical presentations, we postulated a shared underlying etiology. Given the bilateral onset, genetic concordance, and absence of overt systemic symptoms, our workup prioritized etiologies spanning autoimmune disorders, and infectious disease, specifically, Syphilis and Tuberculosis (TB). The systemic work-up was only positive in Twin A, showing high serum ACE, bilateral hilar and mediastinal lymphadenopathy, and skin rash with his previous diagnosis with Celiac Artery Compression Syndrome. Following the exclusion of syphilis and tuberculosis, along with a comprehensive ocular and systemic workup—including pathological examination of a mediastinal lymph node—the collective findings supported a diagnosis of ocular sarcoidosis.

Twin A met the criteria for definite ocular sarcoidosis according to the Revised International Workshop on Ocular Sarcoidosis (IWOS) criteria, as well as the criteria for sarcoidosis-associated uveitis established by the Standardization of Uveitis Nomenclature (SUN) working group [14, 15]. On the contrary, Twin B didn’t even fulfil the criteria for probable ocular sarcoidosis. However, absence of positive laboratory or radiological results, pointed to an underlying immune mediated disease. Yang et al. previously reported a familial case of sarcoidosis associated with uveitis in two sisters [6]. The first sister presented with bilateral panuveitis, while the second exhibited anterior and intermediate uveitis. Both patients had mediastinal lymphadenopathy, and biopsies of the lymph nodes confirmed the diagnosis of sarcoidosis.

One of our patients was previously diagnosed with celiac artery compression syndrome (CAC). Stamler et al. reported an acquired form of CAC syndrome in sarcoidosis, resulting from external compression by enlarged lymph nodes [16]. 

Being monozygotic twins, in addition to the histopathological evidence of non-caseating granuloma, and the skin rash, we considered late onset Blau Syndrome as a possibility. Blau syndrome is an autosomal dominant condition which typically present before the age of 5 with a triad of skin rash, arthritis, and uveitis and is considered as a form of early onset sarcoidosis [17]. Although, our patients were 21 years old, however, minded with their below average IQ, mild symptoms may have passed unnoticed. Sarens et al. conducted a multicenter study investigating ocular manifestations of Blau syndrome [18]. Among 50 patients diagnosed with Blau syndrome, 76% exhibited ocular involvement at baseline. Bilateral uveitis was present in 37 of these 38 patients (97%), while panuveitis affected 51% of the total eyes studied. The most frequent fundus finding was multifocal chorioretinal lesions and no cases of active vasculitis were reported. To the best of our knowledge adult onset Blau like syndrome was reported once by Yao et al. [19]. Unlike, our patients, their patient didn’t show any ocular involvement.

The concurrent bilateral uveitis observed in monozygotic twins, as described in this case, underscores the potential role of genetic predisposition in ocular inflammatory disease and highlights the need to further explore heritable mechanisms in its pathogenesis. Thus, we performed WES; however, the analysis did not identify any causal variants for uveitis, but it did pinpoint a causal variant for their intellectual impairment by identification of a novel, heterozygous likely pathogenic duplication in *SYNGAP1* gene (OMIM # 603384 ) which encodes a ras GTPase-activating protein which is essential for cognition and synapse function [20]. Hence, mutations are responsible for autosomal dominant intellectual developmental disorder-5 (OMIM # 612621) [21]. 

Our inability to detect a clear causal variant for uveitis on WES does not entirely eliminate a genetic etiology, specifically with expansion of the recognized immune disorders. Furthermore, it is possible the culprit variant may be found in a non-coding region undetected by WES.

Though the exact etiology of sarcoidosis remains elusive; however, disease susceptibility is significantly governed by genetic predisposition. Compelling evidence for the presence of a genetic component underlying sarcoidosis has been provided based on familial aggregation, increased risk in siblings and/or relatives, and ability of genome-wide association studies (GWAS), WES or whole-genome sequencing (WGS) studies to identify genetic variants. Proper understanding of this genetic predisposition can enhance a more personalized approach to diagnosis, prognosis and treatment [22]. 

Most variants linked to sarcoidosis risk are immune genes or genes involved in immunologic pathways. HLA region is the most consistently associated region with sarcoidosis risk. Other implicated genes are involved in the immune response as those related to cytokines (*TNF-α* and *IL-23*), cell surface immune receptors (*BTNL2*), macrophage activation (*SLC11A1*) and signaling molecules (*ANXA11*) [22]. 

In addition to genetic predispositions, environmental factors have been implicated in the development of sarcoidosis [23]. Identified triggers include infectious agents (e.g., Propionibacterium acnes, mycobacteria, and fungi), non-infectious organic antigens (such as organic bioaerosols), combustible byproducts, and metal dust [24–27]. The presence of these exposures may explain the simultaneous disease onset in the twins described here. Notably, both individuals had recently begun employment at a garment factory, a setting where occupational exposure to airborne antigens, particulates, or fumes could plausibly contribute to disease initiation.

This case report highlights a rare presentation of sarcoidosis-associated uveitis with simultaneous onset in monozygotic twins, underscoring the potential role of genetic predisposition in disease manifestation. While sarcoidosis is known to cluster in families, synchronous uveitis in twins has never been reported, aligning with prior hypotheses about shared genetic and environmental triggers. This case reinforces the importance of screening siblings of affected patients for early signs of sarcoidosis, particularly in the presence of ocular symptoms. Further research into genetic markers in monozygotic twins could elucidate mechanisms underlying simultaneous disease onset, potentially guiding personalized monitoring strategies for at-risk families.

## Data Availability

No datasets were generated or analysed during the current study.
